# Self-motion with Hearing Impairment and (Directional) Hearing Aids

**DOI:** 10.1177/23312165221078707

**Published:** 2022-03-28

**Authors:** Maartje M. E. Hendrikse, Theda Eichler, Volker Hohmann, Giso Grimm

**Affiliations:** 1 Auditory Signal Processing and Cluster of Excellence “Hearing4all”, Department of Medical Physics and Acoustics, University of Oldenburg, Oldenburg, Germany

**Keywords:** hearing aids, adaptive differential microphone, self-motion

## Abstract

When listening to a sound source in everyday situations, typical movement behavior is highly individual and may not result in the listener directly facing the sound source. Behavioral differences can affect the performance of directional algorithms in hearing aids, as was shown in previous work by using head movement trajectories of normal-hearing (NH) listeners in acoustic simulations for noise-suppression performance predictions. However, the movement behavior of hearing-impaired (HI) listeners with or without hearing aids may differ, and hearing-aid users might adapt their self-motion to improve the performance of directional algorithms. This work investigates the influence of hearing impairment on self-motion, and the interaction of hearing aids with self-motion. In order to do this, the self-motion of three HI participant groups­­­—aided with an adaptive differential microphone (ADM), aided without ADM, and unaided—was measured and compared to previously measured self-motion data from younger and older NH participants. Self-motion was measured in virtual audiovisual environments (VEs) in the laboratory, and the signal-to-noise ratios (SNRs) and SNR improvement of the ADM resulting from the head movements of the participants were estimated using acoustic simulations. HI participants did almost all of the movement with their head and less with their eyes compared to NH participants, which led to a 0.3 dB increase in estimated SNR and to differences in estimated SNR improvement of the ADM. However, the self-motion of the HI participants aided with ADM was similar to that of other HI participants, indicating that the ADM did not cause listeners to adapt their self-motion.

## Introduction

Typical movement behavior in everyday situations can result in the listener not directly facing a sound source when listening to it. This can happen because people do not always look in the direction of interest, and because they do part of the movement with their eyes (Hendrikse et al., 2019a). Moreover, in multi-talker conversations, it is natural to look away from the active speaker from time to time for social reasons (Vertegaal et al., 2001). This movement behavior is known to be highly individual (Kim et al., 2013).

The listener's head orientation influences the SNR at each ear and the performance of directional algorithms in hearing aids because of the head shadow effect (Van Wanrooij & Van Opstal, 2004) and directional pattern of the algorithms. Hendrikse, Grimm, et al. (2020) showed that natural variance in head movements of normal-hearing listeners can result in differences in noise-suppression performance of directional algorithms across individual listeners. Ricketts (2000), Abdipour et al. (2015), and Boyd et al. (2013) have also shown an effect of head movement on the performance of directional algorithms.

It is not yet known whether the movement behavior of hearing-impaired listeners wearing hearing aids differs from the movement behavior of hearing-impaired listeners without hearing aids in everyday listening situations. Previous studies have found that hearing impairment and directional microphones are associated with an increased complexity of orienting behavior towards further off-axis targets (Brimijoin et al., 2010; Brimijoin et al., 2014). Moreover, it has been shown that hearing-impaired listeners with asymmetric hearing loss successfully make use of head movements to increase the level of the target (Brimijoin et al., 2012). This strategy might not maximize the SNR, but it can help to improve it. Grange and Culling (2016) showed that young normal-hearing listeners had difficulty finding a beneficial head orientation. Both of these studies were performed without visual cues. When visual cues are present, listeners are less likely to turn one ear to the target speaker in order to increase SNR (Grange et al., 2018). Moreover, it has been shown that while gaze movements accurately follow the target speaker location, head movements tend to undershoot the target location (Hendrikse et al., 2018; Hendrikse et al., 2019a; Hládek et al., 2019; Lu et al., 2021). Hendrikse et al. (2019a) also found differences between younger and older normal-hearing participants in the relative amount of movement that was done with the head and the eyes (head-eye ratio). Thus, there could also be changes in everyday listening behavior associated with hearing loss and hearing aid use. Based on this literature overview we hypothesize that, like normal-hearing listeners, hearing-impaired listeners and hearing-aid users look at the target speaker. We also expect that hearing-impaired listeners with and without hearing aids orient their head differently, resulting in an increased SNR compared to normal-hearing listeners. If this is the case, the head-eye ratio will differ from that of normal-hearing listeners. In line with this, we expect that participants using a directional algorithm adapt their head orientation to increase the SNR improvement of the directional algorithm.

To test these hypotheses, we investigate how hearing loss and hearing aids with and without a directional algorithm affect self-motion. To identify whether potential differences in self-motion are relevant in the context of hearing research, we also investigate their influence on the signal-to-noise ratio (SNR) and the SNR improvement provided by the directional algorithm. To do this, older hearing-impaired listeners with and without hearing aid experience were recruited. The participants listened to the virtual audiovisual environments (living room, lecture hall, cafeteria, street, and train station) from Hendrikse et al. (2019a), with some small adaptations to adjust the difficulty. Meanwhile, their head, eye and body movements were measured. The hearing loss of the experienced hearing-aid users was compensated using the open master hearing-aid software (openMHA version 4.9.0; Herzke et al., 2017); the participants did not wear their own hearing aids. For half of the experienced hearing-aid users, the sound was processed with a directional algorithm in addition to the hearing loss compensation. An adaptive differential microphone (ADM), after Elko and Pong (1995), was chosen as the directional algorithm, because adaptive differential microphones are often used in commercial hearing aids. Binaural rendering with head-tracking controlled simulation of self-motion was used to render the sound. The self-motion of the participants was analyzed and compared to that of the normal-hearing participants measured in previous work (Hendrikse et al., 2019a). Moreover, the influence of self-motion on the SNR and the SNR improvement by the ADM was analyzed for each participant group using acoustic simulations. Estimating the SNR improvement of the ADM also for the participant groups who did not actually use the ADM allowed us to determine whether the aided participants with ADM adapted their self-motion to improve its performance.

## Method

### Participants

**Figure 1.**
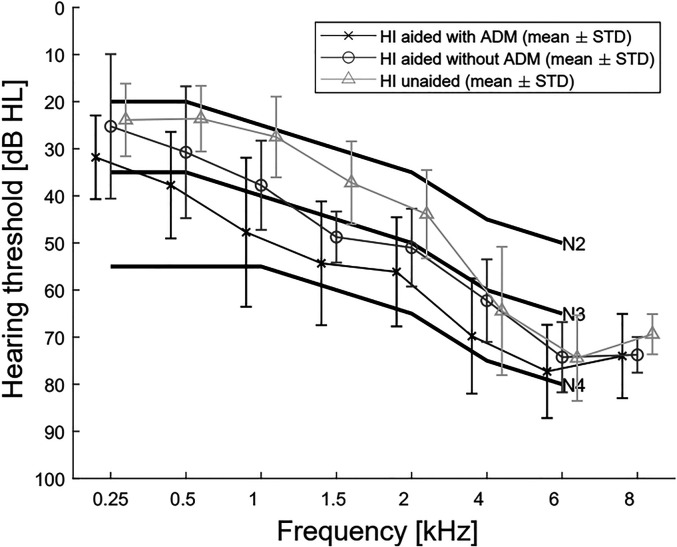
Mean audiograms of the hearing-impaired participants. Plotted are the mean hearing thresholds and standard deviations within each participant group. The N2, N3, and N4 standard audiograms are also included. All hearing thresholds were measured exactly at the frequencies on the X-axis, but the data points were shifted slightly for better visibility.

A total of 30 older (50–80 years, mean age 72.2 years) hearing-impaired (HI) participants were included in this study. The participants were native German speakers and did not suffer from neck or back problems, dizziness or epilepsy. All participants had a moderate, symmetric, sensorineural hearing loss resembling the N3 standard audiogram according to Bisgaard et al. (2010); see Figure 1. The participants were divided into three groups: unaided, aided with ADM, and aided without ADM. In the unaided group, nine participants were included who either did not wear hearing aids in everyday life or had worn bilateral hearing aids for less than two years. The remaining 21 participants had had bilateral hearing aids for more than two years (mean 9.7 years) and wore their hearing aids on average 10.7 h a day. These 21 experienced hearing-aid users were randomly distributed over the aided without ADM (10 participants) and aided with ADM (11 participants) groups. As can be seen in Figure 1, the hearing losses were not entirely balanced between groups. Because the unaided group included inexperienced hearing-aid users, the mean hearing loss was naturally milder. The participants did not wear their own hearing aids during the experiment; the hearing loss compensation and processing with ADM were done using the openMHA, as described in the setup section. The participants were not selected based on visual acuity, but all participants wore glasses.

### Environments and Tasks

The task of the participants during the experiment was to listen to, and look at, a number of virtual audiovisual environments (VEs) representing situations from everyday life, while their self-motion was recorded. In each VE, a target was defined that the participants had to attend to; each VE was presented once to each participant. For recording participants’ self-motion, it was important that they made an effort to understand the target and did not give up. For this reason, the SNR of the VEs was adjusted individually. For the individual adjustment, each individual's 70% speech reception threshold (SRT70) was measured using the Oldenburg matrix sentence test (OLSA; Wagener et al., 1999) in the diffuse background noise of the cafeteria. The mean SRT70 of 10 NH participants was subtracted from the individual SRT70 and the overall noise level was reduced by this difference. This way, the noise level was on average reduced by 6.8 ± 2.5 dB for the HI participants. The individual SNR adjustment reduced the ecological validity of the VEs, but the aim was to equalize the intelligibility of each VE across participants to make sure that the task was neither impossible nor too easy. As explained in the analysis section, the acoustic simulations only used the self-motion data, and the fixed, more ecologically valid SNRs from the previous study could be used for all datasets.

Before the actual measurement, the participants could listen to each VE (without target) for about 30 s to become familiar with the environment (the VEs had a duration of 88 to 144 s). To encourage participants to pay attention to the target, they were asked to fill in a content-based multiple-choice questionnaire after each VE; the responses were not evaluated. Some of the VEs were presented while the participants were seated, and some while they were standing, as indicated below; the order was randomized. When standing, the participants were told to stay close to the center of the setup, so that they did not leave the range of the sensors. The experimental procedure was approved by the ethics committee of the Carl von Ossietzky University of Oldenburg.

The VEs from Hendrikse et al. (2019a; 2019b) were used in this study, with a few small adaptations. These VEs represented the following scenarios:

1. Listening to a multi-person conversation in a cafeteria.
2. Listening to a lecture in a lecture hall.
3. Listening to the news in a living room.
4. Listening to announcements in a train station.
5. Listening to a multi-person conversation at a bus stop on a street (hereafter: street_active_).
6. Waiting at an intersection (hereafter: street_passive_). This was a passive listening situation, i.e., there was no defined target.

**Figure 2.**
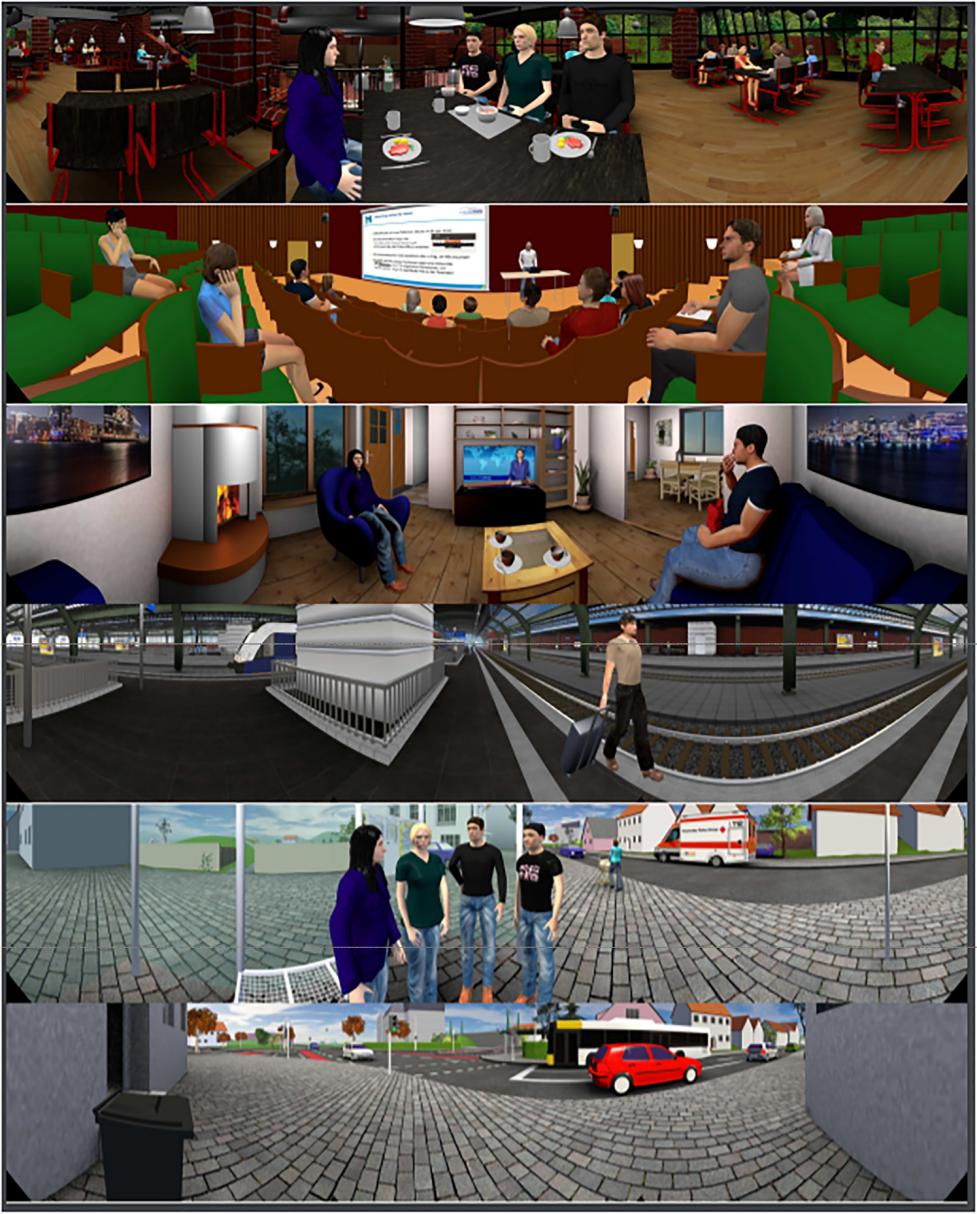
Pictures of the virtual audiovisual environments (VEs) from Hendrikse et al. (2019a, 2019b). From top to bottom: cafeteria, lecture hall, living room, train station, street_active, and street_passive.

In the cafeteria, lecture hall, and living room VEs, the participants were seated; in the train station and street VEs, the participants were standing. The cafeteria VE was presented twice, once each with a different target conversation. During the first presentation (hereafter: cafeteria_listeningonly_ VE) the task of the participant was to follow the conversation between the animated persons at the table. The second presentation (hereafter: cafeteria_dualtask_ VE) also tested hand-eye coordination. The subjects were asked to follow the conversation and, at the same time, insert one pin after the other into the holes of a Purdue Pegboard (Tiffin & Asher, 1948). The purpose of this dual task was to mimic the everyday situation of eating and listening to a conversation at the same time. In this kind of situation, which is common in a cafeteria, the self-motion would be different than when a person is listening only. A more detailed description of the VEs, including presentation levels, can be found in Appendix A. Sample pictures are shown in Figure 2.

Hendrikse et al. (2019a) concluded that these VEs were sufficiently realistic, but they made some suggestions for improvement based on the comments of the participants. Most importantly, the participants found the conversations in the cafeteria VE unrealistic, because no Lombard speech was included in these conversations. The Lombard effect is defined as the tendency of speakers to increase their vocal effort, e.g. change in loudness, pitch, rate and duration of syllables, when speaking in loud noise in order to increase their intelligibility (Fitzpatrick et al., 2015; Garnier et al., 2010; Lu & Cooke, 2008). Without Lombard speech, the conversations were too hard to understand in such a noisy environment despite the realistic SNR. Therefore, new conversations were recorded and implemented including Lombard speech, with three actors (Gerken et al., 2020). To achieve this, the three actors in the conversations were listening to cafeteria noise via headphones while their speech was recorded. Because the conversations in the cafeteria VEs were exchanged, the multiple-choice questions were also changed with respect to the previous study.

### Setup

This section explains how the virtual audiovisual environments were presented to the participants, and how the self-motion was measured. The measurements were conducted in the audio lab in Oldenburg; for illustrations see Hendrikse et al. (2018). The setup was placed in a sound-treated room, and the equipment was attached to a cloth-covered metal frame, with a diameter of 3.5 m, that reduced environmental sounds, light, and room reflections.

#### Visual Presentation

The visual stimuli were projected onto a cylindrical, acoustically transparent screen using three projectors (NEC U321H). The projected arc encompassed 300°. A graphics card (Nvidia Quadro m5000) performed the warping necessary for projecting onto a cylindrical screen. This process was calibrated manually. The virtual visual environments were created in Blender (version 2.78a; Roosendaal, 1995) and were rendered using the built-in game engine of this software package. A simulation of movement parallax was added to potentially increase the presence and involvement of the participants. This changed the visual perspective according to the small sways and slight translations the participants made. The position of the virtual camera and the virtual listening position (see next section) were displaced by half of the physical displacement of the head to account for the displacement relative to the projection screen and loudspeakers. That is, a head translation of 10 cm would be equivalent to a camera/listening position translation of 5 cm in the virtual environments.

#### Audio Presentation

The virtual acoustic environments were created using the software package TASCAR (version 0.202-0.204; Grimm et al., 2019). The overall noise level in all VEs was adjusted individually according to the SRT70 scores of the participants, as described above in the ‘Environments and tasks’ section; for presentation levels see Appendix A. In the current study, the signals were played back over headphones (Sennheiser HDA 200). The free-field response was derived from ISO 389-8 (2004) and compensated accordingly. After compensation, the broadband output level was calibrated using an artificial ear (Brüel & Kjær type 4153) using a noise stimulus with average speech power spectral density. For headphone playback, rendered signals were convolved with previously measured head- and hearing-aid-related impulse responses (HARIRs; in-ear, plus three hearing aid microphone positions on each side). The signals were rendered for a loudspeaker layout of 29 loudspeakers, which matched the layout used for recording the HARIRs. The rendering for the loudspeaker layout calculated how much energy came from each direction for each source, which was needed before the HARIRs could be applied. The loudspeakers were arranged in a 16-loudspeaker horizontal ring array with 1.76 m radius at ear level (first loudspeaker at 11.25° from frontal direction, with 22.5° spacing), two 6-loudspeaker ring arrays at + 45° and −45° elevation (first loudspeakers at 0° and 30° azimuths from frontal direction, respectively, with 60° spacing), and one pole loudspeaker (90° elevation, directly above the participant). The HARIRs were recorded as described by Grimm et al. (2020) using a head-and-torso simulator (Brüel & Kjær Type 4128C with artificial ears: 4158C right and 4159C left, pre-amplifier 2669) with behind-the-ear hearing aid dummies with three microphones on each side, so they were non-individualized.

A preliminary version of the recordings by Grimm et al. (2020), which contained an error in the channel order for the upper and lower loudspeaker rings, was used during the measurements in this study. The influence of this error on self-motion was assessed in an additional experiment (Appendix B), by comparing with the final HARIRs reported by Grimm et al. (2020). The effect of the error on self-motion was found to be negligible (p > 0.05, effect sizes ≤ 0.3).

After convolution, the resulting signals for the front hearing aid microphones were played back over headphones for the unaided group of participants. The HARIRs from the front hearing aid microphones were used with the unaided group so that they would be more comparable with the aided groups. For the aided group without ADM, the HARIRs from the front hearing aid microphones were also used, and hearing loss compensation was applied. For the aided group with ADM, the signals resulting from convolution with HARIRs from front and rear hearing aid microphones were used as input for the ADM, and hearing loss compensation was applied to the ADM output. Moreover, the head translations in the horizontal plane and the head yaw measured by the head movement sensor were sent to TASCAR, and the position of the acoustic receiver adjusted accordingly, so that the presented audio changed according to the head movements the participants made (binaural rendering with head-tracking controlled simulation of self-motion). The audio was processed with a block size of 1024 samples at a sampling rate of 44100 Hz. The latency between the head movements of the participants and the adjustment of the binaural rendering based on those movements was on the order of 40 ms.

#### Hearing Loss Compensation and ADM

The hearing loss compensation and ADM were applied using the open master hearing-aid software (openMHA version 4.9.0; Herzke et al., 2017; Kayser et al., 2021). The hearing loss compensation was performed based on the measured audiogram of the participants, using the CR2 NAL-RP fitting rule. CR2 NAL-RP is based on the linear NAL-RP fitting rule developed by the National Acoustics Laboratories (Byrne et al., 1990). In addition, it adds a compression of 2:1 with a knee point at the narrow-band levels corresponding to 40 dB SPL long term average speech spectrum (LTASS; Byrne et al., 1994). The gain was adjusted to reach the NAL-RP prescription at 65 dB SPL LTASS. Below the knee point the amplification was kept constant to prevent extreme amplification of soft sounds. A hard limiter was added to prevent presentation levels above 105 dB SPL.

The implementation of the ADM in the openMHA is based on the algorithm described by Elko and Pong (1995). The ADM is a monaural algorithm using a single pair of omnidirectional microphones. It generates front-facing and back-facing cardioid patterns by subtracting the microphone signals from each other after delaying one signal by the amount of time it takes sound to travel the microphone distance. The front-facing and back-facing cardioid patterns were combined using a mixing weight. By adapting the mixing weight, a spatial zero was steered towards the most prominent sound source in the back hemisphere, as the target was assumed to be in front. Two separate ADMs were used for the left and right sides. The signal was processed with the ADM before applying the hearing loss compensation.

#### Sensors

Six infrared cameras (Qualisys Miqus M3) were used to measure the head and torso movements. These tracked the subjects’ movements via highly reflective markers attached to the headphones (head movement), and the right and left shoulder pads of a vest (torso movement). The three axes of rotation were recorded—roll, pitch and yaw—as well as the translation on these axes. The motion was recorded by the motion tracking software Qualisys (version: 2019.3.4780) with a sampling frequency of 200 Hz.

Eye movements were measured by a custom-made wireless electrooculogram sensor (EOG). One electrode was placed on the temple next to each eye. The measured signal was sent via WLAN with a sampling rate of 33 Hz and a resolution of 16 bits. Thus, eye movements in the horizontal plane could be measured with an accuracy of approximately ± 10°. After measurement, the drift in the eye movement data was approximated by linearly extrapolating the data and then smoothing with a moving-average filter with a length of 500 samples. This approximated drift was subtracted from the eye movement data.

The calibration of the sensors was done using a cross projected onto the screen. To align the head movement sensor with the head direction, the participants were asked to adjust the markers on the headphones until the cross was placed as accurately as possible in front of their nose. To calibrate the EOG sensor, the cross was displaced 5–30° to the left or right. The participants were asked to follow the cross with their eyes; accidental head movements were compensated for. After switching between the standing and sitting environments, calibration was repeated. To obtain the gaze trajectories, the head- and eye-movement trajectories were resampled to the same time-line with a 120-Hz sampling rate and summed. The TASCAR and LabStreamingLayer (Medine, 2016) software packages were used for time synchronization and data logging.

### Analyses

#### Analysis of Self-Motion

To analyze how hearing impairment and hearing aids with and without a directional algorithm affect self-motion, the self-motion of the three HI participant groups—unaided, aided without ADM and aided with ADM—was compared. The movement measures used by Hendrikse et al. (2019a) were calculated. These include the standard deviation of the gaze trajectories (GazeStd); the standard deviation of the head trajectories (HeadStd); the mean speed of the gaze trajectories (GazeSpeedMean); the mean speed of the head trajectories (HeadSpeedMean); the number of gaze jumps, normalized by the duration of the VE (NGazeJumps); and the absolute head angle relative to torso over the absolute gaze angle relative to torso (HeadGazeRatio). For calculating the HeadGazeRatio, there were three criteria for excluding certain data points: (a) if the time point was during a head/eye saccade; (b) if the head angle was bigger than the gaze angle, or of opposite sign; and (c) if the gaze angle was smaller than 10°. Data points during head/eye movement were excluded because we were interested in the static situation; the head and eyes do not move at the same time or speed and including these data points would result in a large spread of ratio values. The second exclusion criterion was applied because it was unclear what the ratio would represent in such a situation. The third exclusion criterion was applied to avoid division by zero and because the values for the ratio in this range would be determined mostly by the sensor noise. On average, 65% of the data points were excluded, and in four cases, the HeadGazeRatio measure could not be calculated for a participant in one VE.

The root-mean-squared (RMS) error between the direction of the target and the head (TargetHeadRMS) or gaze direction (TargetGazeRMS) was calculated to quantify the deviation from the target direction in a scalar metric. In the VEs with multi-talker conversations, the direction of the active speaker at each time point was taken as the target direction, and in the lecture hall it was the direction of the lecturer. In the train station and street_passive_ VEs, no target direction was defined, so the TargetHeadRMS and TargetGazeRMS were not calculated in these VEs.

#### Acoustic Simulations

The acoustic simulation method presented by Hendrikse, Schwarte, et al. (2020) was used to estimate the SNRs resulting from the individual head movement trajectories of the HI participants in the VEs. In the acoustic simulations, the rendered signals for the 29-loudspeaker layout, as described in the ‘Audio presentation’ section, were effectively rotated and shifted to match the measured head yaw and head translations of the participants. This resulted again in 29 channel loudspeaker signals, now including the movement. Then, the signals were convolved with the final HARIRs from Grimm et al. (2020), resulting in hearing aid microphone signals. The acoustic simulations were done separately for the target and noise signals, so that the SNR could be calculated. From the hearing aid microphone signals, the better-ear SNR was calculated using segmental SNR after Quackenbush et al. (1988), with 200-ms non-overlapping windows. By comparing these SNRs between participant groups, differences might become apparent that were not visible in the analysis of self-motion, enabling us to analyze whether differences are relevant for assessing acoustic communication ability.

Because only the measured self-motion of the participants was used in the acoustic simulations, the noise level of the VEs could be set to any value. No noise level reduction was applied in the acoustic simulations, not only ensuring a higher ecological validity, but also making the results comparable by having the same level for all participants. The signals resulting from the acoustic simulation were thus very similar to the signals that were presented to the participants during the experiment, except that no noise level reduction was applied and that the head yaw and head translations were applied to the rendered loudspeaker signals instead of to the acoustic scene directly. This was done in line with the method described by Hendrikse, Schwarte, et al. (2020).

In addition, the hearing aid microphone signals resulting from the acoustic simulations were processed with the ADM using the openMHA. To be able to calculate the SNR after processing with the ADM, the method described by Hagerman and Olofsson (2004) was applied. The better-ear SNR was calculated from the processed signals as described in the previous paragraph. In order to analyze how the ADM would have improved the SNR for the different head movement trajectories, the window-wise difference between the SNRs of the hearing aid microphone signals processed with the ADM and the unprocessed signals for each head movement trajectory were taken and then averaged over the entire trajectory for the statistical analysis (SNRimprovementADM). Because the acoustic simulations were done separately from the recording of the self-motion, this could be done for the head movement trajectories of all participants, regardless of their aided condition when recording the self-motion. Estimating the SNR improvement of the ADM for the participant groups who did not actually use the ADM allowed us to determine whether the aided participants with ADM adapted their self-motion to improve its performance. Since no target source was defined in the street_passive_ VE, the SNR measures could not be calculated there.

#### Data from Previous Study

The previously recorded self-motion data of 21 younger normal-hearing (NH) and 19 older NH participants were also included in the analyses, and are available online (Hendrikse et al., 2019c). The self-motion of the NH participants was also measured in the previously mentioned VEs, but at a different SNR than the self-motion of the HI participants. Only the cafeteria VEs were different, using three-talker conversations with Lombard speech instead of the four-talker conversations without Lombard speech in the previous study. Another difference is that the VEs were presented to the NH participants via loudspeakers, and to the HI participants over headphones. The influence of wearing headphones on self-motion was assessed in an additional experiment by comparing with presentation via loudspeakers (Appendix B) in order to ensure that a comparison between the NH and HI self-motion data is valid. No significant differences in self-motion were found between headphone and loudspeaker presentation for the measures from the ‘Analysis of self-motion’ section (p > 0.05, effect sizes ≤ 0.3). These measures were also calculated for the self-motion data of the NH participants.

The acoustic simulations were also done with the self-motion data of the NH participants. This was done with the same recordings of the VEs, with the exception of the cafeteria VEs, where the target conversation was different. To enable comparison in the cafeteria VEs as well, the SNRs without movement (always facing frontal direction) for the VEs were taken as a reference (Table 1), and the SNRs including the head movement of the HI and NH participants were calculated relative to this reference. Window-wise differences were taken, and then averaged over the entire head trajectory for the statistical analysis (SNRrelative). The SNRimprovementADM data for the NH participants was also calculated and included in the comparison.

**Table 1.** Reference SNRs Without Movement (Always Facing Frontal Direction) for Each Participant Group.

cafeteria_dualtask_	cafeteria_listeningonly_	lecture hall	living room	train station	street_active_
NH: 1.7 dB HI: −1.5 dB	NH: −1.2 dB HI: −3.1 dB	4.9 dB	6.5 dB	−7.0 dB	3.8 dB

#### Statistics

A multiway ANOVA was done for the movement measures GazeStd, HeadStd, GazeSpeedMean, HeadSpeedMean, NGazeJumps and HeadGazeRatio. The participant group (unaided, aided without ADM, aided with ADM, and the younger NH and older NH participants from previous work) was included as a between-participants factor. In previous work, a significant effect of the different VEs on self-motion was found, so the environment was included as a within-participant factor, but only to check for interaction effects with the participant group. Similarly, two separate multiway ANOVAs were done for the TargetHeadRMS and TargetGazeRMS measures, and the SNR measures SNRrelative and SNRimprovementADM, but without the train station and/or street_passive_ VEs, because these measures could not be calculated there, as described before. To correct for multiple comparisons (Cramer et al., 2016), the false discovery rate was controlled with the Benjamini-Hochberg procedure (Benjamini & Hochberg, 1995) for the grouped results of all ANOVAs. The Greenhouse-Geisser correction was applied when the sphericity assumption was violated.

## Results

### Analysis of Self-Motion

The main effects of the multiway ANOVAs for the movement measures are shown in Table 2. There was a strong effect of participant group on HeadGazeRatio, and a weaker effect on GazeStd. Moreover, there were weak interaction effects between participant group and environment for all measures except HeadSpeedMean. In the following, the measures that were significantly affected by participant group are analyzed in more detail. Because all of these measures also had significant interaction effects, pairwise comparisons (with Bonferroni correction) were done to check for differences between participant groups in each VE.

**Table 2.** Main Effects of Multiway ANOVAs for the Movement Measures.

Factor	Measure	F-value	p	p_adj_	Effect Size
group	GazeStd	F(4,63) = 3.2	0.018	0.033*	0.18
	HeadStd	F(4,63) = 2.2	0.081	0.095	0.13
	GazeSpeedMean	F(4,63) = 2.6	0.045	0.059	0.15
	HeadSpeedMean	F(4,63) = 2.1	0.089	0.098	0.13
	NGazeJumps	F(4,63) = 2.4	0.062	0.079	0.14
	HeadGazeRatio	F(4,63) = 138.6	<0.001	<0.001***	0.91
	TargetGazeRMS	F(4,63) = 4.3	0.036	0.056	0.22
	TargetHeadRMS	F(4,63) = 2.7	0.612	0.612	0.15
group* environment	GazeStd	F(15.8,229,5) = 3.4	<0.001	<0.001***	0.19
	HeadStd	F(12.5,180.6) = 2.1	0.019	0.032*	0.13
	GazeSpeedMean	F(16.3,236.0) = 3.7	<0.001	<0.001***	0.20
	HeadSpeedMean	F(10.5,152.0) = 2.0	0.036	0.052	0.12
	NGazeJumps	F(19.3,280.3) = 4.0	<0.001	<0.001***	0.22
	HeadGazeRatio	F(17.9,259.7) = 2.3	0.003	0.006**	0.14
	TargetGazeRMS	F(11.1,175.6) = 3.2	<0.001	0.001**	0.17
	TargetHeadRMS	F(9.7,152.8) = 3.8	<0.001	<0.001***	0.20

*Note.* The p_adj_-column lists p-values adjusted for multiple comparisons (Benjamini-Hochberg procedure). Significances are indicated at the *0.05 level **0.01 level ***0.001 level.

**Figure 3.**
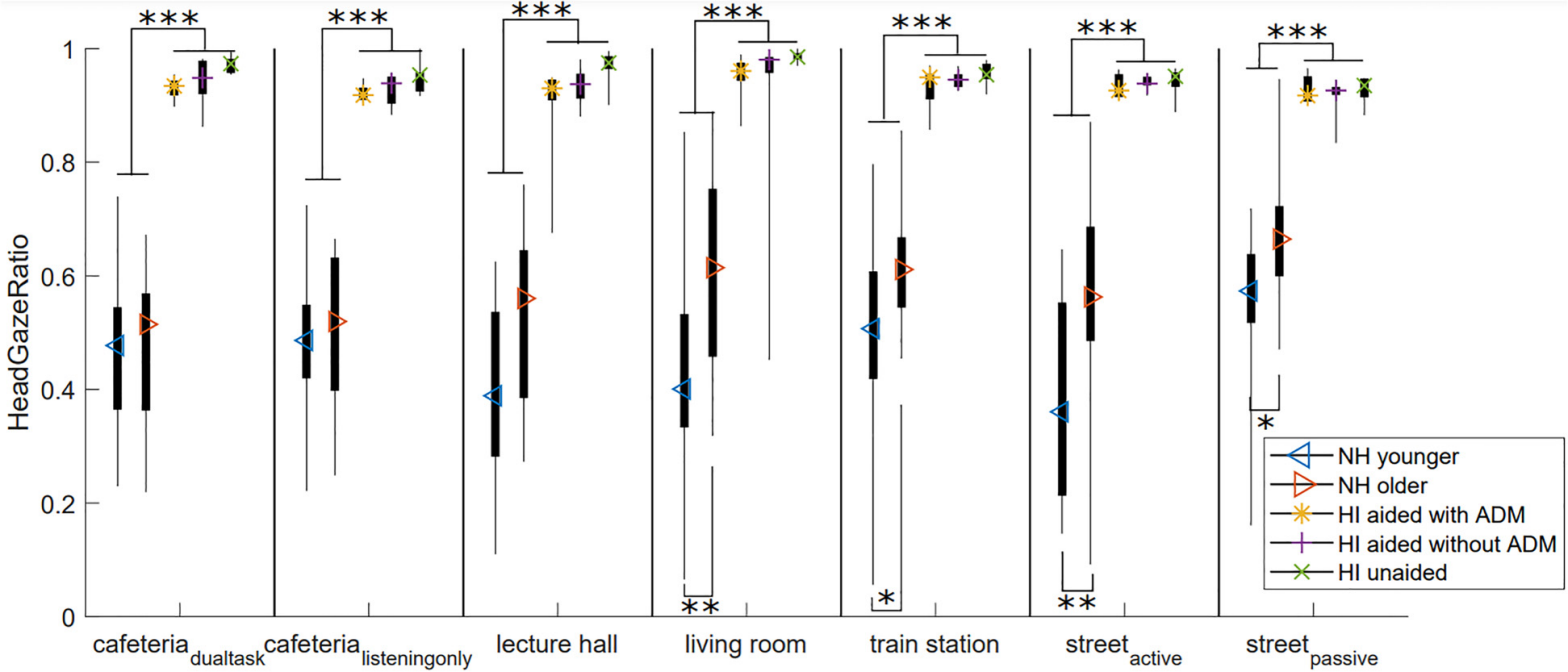
Boxplots of the headGazeRatio (1 = all head movement, 0 = all eye movement) for all participant groups in all VEs. Significant differences are indicated at the *0.05 level, **0.01 level, ***0.001 level. Boxplots show the range (thin line), 25th and 75th percentiles (thick line) and the median (colored symbol).

To look into the significant effect of participant group on HeadGazeRatio, HeadGazeRatio is shown in Figure 3 per participant group and VE, and significant differences according to pairwise comparisons are indicated. Overall, HI participants had a significantly higher HeadGazeRatio than NH participants (p < 0.001), indicating that they did relatively more of the movement with their head, and less with their eyes compared to NH participants. In fact, the HeadGazeRatio of the HI participants was almost 1 (mean 0.94 ± 0.05), indicating that they did almost all of the movement with their head. This can have to do with age, since the older NH participants had significantly higher HeadGazeRatios than the younger NH participants in most of the VEs; but it is probably related primarily to hearing impairment. Although the HI participants did more of the movement with their head than the NH participants, this did not result in a more accurate orientation of their head towards the target direction (no significant participant group differences in TargetHeadRMS).

**Figure 4.**
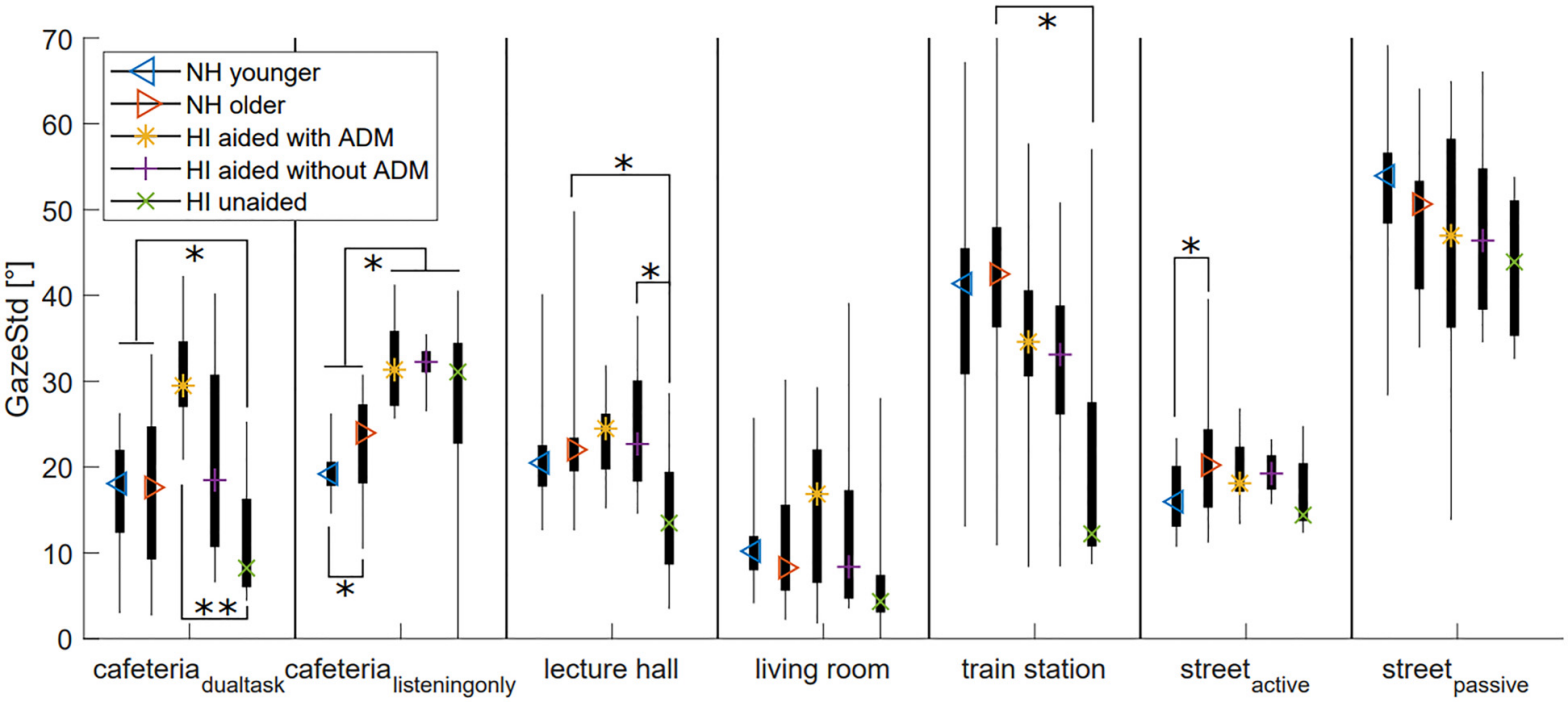
Boxplots of the gazeStd for all participant groups in all VEs. Significant differences are indicated at the *0.05 level, **0.01 level. Boxplots show the range (thin line), 25th and 75th percentiles (thick line) and the median (colored symbol).

The same was done for the GazeStd (Figure 4). Overall, the unaided participants had a significantly lower GazeStd (p = 0.046) than the older NH participants, which can be seen in some, but not all, of the VEs in Figure 4. Thus, the unaided participants made slightly fewer or smaller gaze movements than the older NH participants.

### Acoustic Simulations

**Figure 5.**
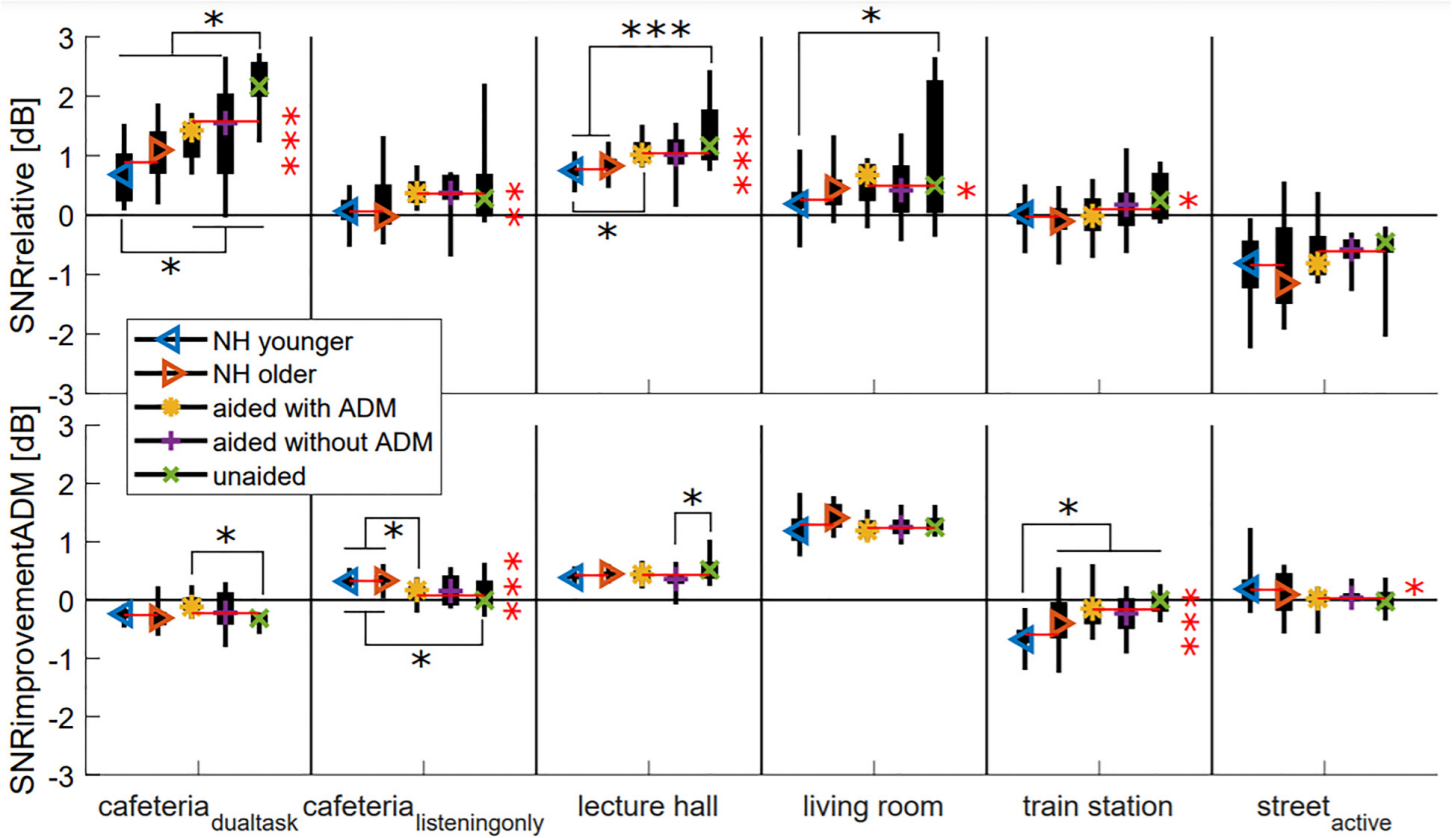
The SNR resulting from the head movement of the participants, relative to the SNR without movement (always facing frontal direction), for all participant groups in all environments (top). The SNR improvement provided by the ADM for the head movement of the participants (bottom). Significant differences are indicated at the *0.05 level, **0.01 level, ***0.001 level. Boxplots show the range (thin line), 25th and 75th percentiles (thick line) and the median (colored symbol). The group median of the normal-hearing and hearing-impaired participants (horizontal lines), and significant group differences (asterisks) are indicated in red.

The outcomes of the multiway ANOVA that was performed on the SNR measures calculated from the acoustic simulations are displayed in Table 3. There was a moderate effect of participant group on SNRrelative. Furthermore, there were weak interaction effects of participant group and environment on SNRrelative and SNRimprovementADM. SNRrelative and SNRimprovementADM are plotted in Figure 5 per participant group and VE. Because the interaction effect was significant for both measures, pairwise comparisons (with Bonferroni correction) were done between participant groups in each VE, and significant differences are indicated.

**Table 3.** Main Effects of Multiway ANOVA for the SNR Measures.

Factor	Measure	F-value	p	p_adj_	Effect Size
group	SNRrelative	F(4,65) = 12.2	<0.001	<0.001***	0.43
	SNRimprovementADM	F(4,65) = 1.5	0.221	0.233	0.08
group*environment	SNRrelative	F(14.6,237.4) = 2.1	0.014	0.027*	0.11
	SNRimprovementADM	F(14.8,240.6) = 4.5	<0.001	<0.001***	0.22

*Note.* The p_adj_-column lists p-values adjusted for multiple comparisons (Benjamini-Hochberg procedure). Significances are indicated at the *0.05 level **0.01 level ***0.001 level.

Overall, the HI participants had a significantly higher SNRrelative (0.5 dB; 0.4–0.6 dB 95% CI) than the NH participants (0.2 dB; 0.1–0.3 dB 95% CI; p < 0.001). This occurred in all VEs (p < 0.05) except the street_active_ VE (p = 0.089). Moreover, the unaided HI participants had a higher SNRrelative than the other HI participants (p < 0.05), but the difference was only significant in the cafeteria_dualtask_ VE. This indicates that the head movements of all participant groups increased the SNR compared to the situation without head movement (always facing frontal direction), but the self-motion of the HI participant groups led to the highest SNR.

The NH participants had a significantly higher SNRimprovementADM than the HI participants in the cafeteria_listeningonly_ and street_active_ VEs, but a significantly lower SNRimprovementADM in the train station VE. These differences were small, at most 0.3 dB. Therefore, there was no overall effect of participant group on SNRimprovementADM. Moreover, no significant differences in SNRimprovementADM were found between the head movement trajectories of the HI participants that were aided with the ADM and the HI participants that were aided without ADM. The unaided HI participants had a significantly lower SNRimprovementADM than the HI participants aided with ADM in the cafeteria_dualtask_ VE, but there were no significant differences in the other VEs. The SNRimprovementADM was significantly higher than zero on average (0.2 dB; 0.2–0.3 dB 95% CI), but the ADM provided no significant SNR improvement in the cafeteria_dualtask_ and train station VEs.

## Discussion

The analysis of self-motion shows that, as expected, both the HI and the NH participants looked at the target speaker. The gaze-related movement measures (GazeStd, GazeSpeedMean, NGazeJumps, TargetGazeRMS) showed no, or only a small, group effect. This could indicate that the participant groups had a similar gaze-related self-motion, or that the measures are not sensitive enough to detect differences. In accordance with the results of Lu et al. (2021) and Hládek et al. (2019), the participants used a combination of head and eye movements to look at the target. The significant differences in HeadGazeRatio that were found between the NH and HI participant groups indicate that the HI participants covered a larger part of the movement with head turns compared to NH participants. Within the NH participants, the older participants covered more of the movement with head turns than did the younger participants, although this had a much smaller effect size. As noted in previous work (Hendrikse et al., 2019a), wearing glasses also had a small effect (η^2^ of 0.18) on the HeadGazeRatio, probably because glasses limit the maximum eye angle, leading to a higher HeadGazeRatio. Because all HI participants were wearing glasses, the effect of wearing glasses could not be tested within the HI participants, but it could have had a modest contribution to group differences. Thus, group differences are mainly related to hearing impairment, potentially with minor contributions of age and wearing glasses.

Lu et al. (2021) did not find significant differences related to age or hearing impairment in the relative amount of movement covered with head and eye turns, but they did report a trend in the same direction as in the current study. In their study, Lu et al. (2021) measured fewer participants (ten younger NH, ten older NH, eight older HI), which could explain why the differences were not significant. Moreover, all participants in the Lu et al. (2021) study wore eye tracker glasses, which limit the maximum eye angle in a similar way as regular prescription glasses. This means that, unlike in the current study, wearing prescription glasses could not have contributed to group differences in the Lu et al. (2021) study.

The values for the HeadGazeRatio found for the HI participants differed somewhat from the values reported by the other two studies, where participants covered about 50% (Hládek et al., 2019) or 50–80% (Lu et al., 2021) of the movement with head turns for targets located at 30° off-axis. There are, however, also some differences in the calculation method. For example, for the calculation of the HeadGazeRatio measure, we decided here to exclude data points measured during head/eye saccades, because head and eye saccades take place with different timing and speed and would thus add noise to the measure, as we are mainly interested in the final orientation.

The increased HeadGazeRatio of the HI participants did not result in a more accurate orientation of the head towards the target source (no effect of participant group on TargetHeadRMS); this would be expected if both NH and HI participant groups looked at the target position. Nor was there an effect of participant group on HeadStd or HeadSpeedMean. Apparently differences did not occur so much during head/eye saccades, but only for the final orientation (only these data points were included in the HeadGazeRatio). As shown by Brimijoin et al. (2012) and Grange et al. (2018), pointing the head exactly at the target source may not be the optimal orientation to increase the (better-ear) SNR, but rather, depending on the position of the noise source(s), orienting somewhat off-axis may be better. The acoustic simulations were therefore carried out to assess the effect of self-motion on the SNR. SNRs resulting from self-motion of HI participants were significantly higher than those of NH participants, but only by 0.3 dB on average. In both groups, self-motion increased the SNR significantly compared to keeping the head pointed in the frontal direction, but this was also a small effect (0.2 dB and 0.5 dB on average for NH and HI participants, respectively). The fact that there were significant differences in the SNR, but not in the head-related movement measures indicates that the latter may not be sensitive enough, but they were shown to be sensitive in previous work (Hendrikse et al., 2019a).

The range in SNRrelative across participants was larger: up to 3 dB averaged over the duration of the VEs. Moreover, the reported values are the average over the duration of the VEs, and the differences at single time points could be larger. It would be interesting to see what SNRrelative could be reached when optimizing the head movement. Finding the optimum would be difficult due to the dynamic nature of the VEs, and due to limitations in consecutive orientations related to physical limitations of head movement. However, self-motion strategies to optimize the SNR in more realistic environments would be an interesting topic for future research. Moreover, instead of the broadband SNR, speech intelligibility models could be applied to the data to give a more accurate prediction of the speech intelligibility benefit.

The different groups of HI participants had very similar self-motions. This suggests that the aided participants with ADM did not adapt their self-motion to increase the SNR improvement of the ADM. This is in line with the results of Hládek et al. (2019), who also found no adaptation of the head-orientation strategy related to the use of directional algorithms. Brimijoin et al. (2014) did find an effect of hearing-aid directionality on how fast listeners can orient towards an auditory-only target; but that may have been due to that study being solely an orientation task and/or its lack of visual reinforcement of the target location. In the study of Hládek et al. (2019), the task was speech recognition, and there was visual reinforcement of the target location (a static facial image). To further investigate the adaptation of self-motion, the SNR improvement of the ADM that would have resulted from participants’ head movement was calculated for all participant groups. The SNRimprovementADM for the aided participants with ADM was indeed not significantly different from that for the aided participants without ADM. Compared to the NH participants, the HI participants had a significantly higher SNRimprovementADM in the train station VE, but a significantly lower SNRimprovementADM in the cafeteria_listeningonly_ and street_active_ VEs. These differences were small: only 0.3 dB at most. The ADM provided a small but significant SNR improvement of 0.2 dB on average, and no significant improvement in the cafeteria_dualtask_ and train station VEs. Such a small benefit of the ADM may not have been enough of an incentive to adapt the self-motion. This ADM implementation has previously been shown to provide up to 15 dB SNR improvement, although in an anechoic situation with only one noise source (Hendrikse, Grimm, et al., 2020).

The aided participants with ADM had a directional mode in their own devices, and they were allowed to briefly get used to the VE and the ADM output before the measurement started. However, it is possible that more time is needed to get accustomed to the ADM before users begin to adapt their self-motion. It was not possible for us to explore this question, because the ‘hearing aid’ in this study was implemented using the openMHA on a PC. Recent developments in hardware have resulted in a portable device running the openMHA (Herzke et al., 2018; Kayser et al., 2021; Pavlovic et al., 2019), so it is now possible to conduct a more longitudinal study design where the participants can get accustomed to the HA algorithm(s) at home. It would then also be possible, and interesting, to measure the effect of other algorithms, for example a narrower beamformer, on self-motion. This kind of algorithm is different from what participants are used to with their own devices, and would definitely require a longer time period to get accustomed to. A narrower beamformer may affect head movement more, because it has a stronger attenuation of lateral sound sources. Moreover, it could potentially provide a larger benefit, which would be a stronger incentive to actually adapt self-motion.

## Conclusion

Hearing impairment had a strong effect on the relative amount of head and eye movement (head-eye ratio). The HI participants did almost all movement with their head, whereas the NH participants did relatively more of the movement with their eyes. As shown in previous work, age and wearing glasses had a smaller effect on the head-eye ratio, so these factors could have had a minor contribution to group differences. The different groups of HI participants had very similar self-motion. The self-motion of the HI participants resulted in a slightly increased SNR, which was estimated using acoustic simulations, both in comparison with the NH participants (0.3 dB difference) and in comparison with the situation where the head always faced the frontal direction (0.5 dB difference).

The SNR improvement provided by an adaptive differential microphone (ADM) was estimated using acoustic simulations, also for movement trajectories of the participants who were not aided with the ADM. There were some small (<0.4 dB) significant differences in SNR improvement by the ADM between participant groups in some virtual environments, but no overall effect. The estimated SNR improvement was not significantly different for the movement trajectories of the participants aided with ADM compared to the HI participants that were not aided with the ADM during the measurements. This is in line with the finding that the self-motion was very similar for the HI participant groups. It can be concluded that the participants aided with ADM did not adapt their self-motion to increase the benefit of the ADM. It would be interesting to test the interaction of different algorithms, for example narrower beamformers, with self-motion in the future. A narrower beamformer may affect head movement more, because it provides a stronger attenuation of lateral sound sources. Moreover, it potentially provides a larger benefit, which would be a stronger incentive to actually adapt the self-motion.

## Appendix A

### Descriptions of Virtual Audiovisual Environments

A brief description of the virtual audiovisual environments is given here, as well as the presentation levels measured when presenting the virtual environments over loudspeakers. The noise level listed here is the level that was used in the previous study with NH participants. For the HI participants in the current study, the noise level was individually adjusted based on the SRT70 measurement, resulting in a noise level reduction of 6.8 ± 2.5 dB on average. Further acoustic features can be found in Hendrikse et al. (2019a).

In the living room VE, the participant sat on a sofa in the virtual living room with a person eating chips (-90°; negative angles to the right, 0° in frontal direction) next to him. A person commenting on the news (45°) sat on an armchair, and in the background a fire crackled. The participants were asked to attend to the newsreader on the TV set in front (-4°). Through the open door to the kitchen, noises of the dishwasher, water kettle and fridge could be faintly heard. Target sound level L_eq_: 60.7 dBA, noise sound level L_eq_: 52.5 dBA.

In the lecture hall VE, the participant sat in the audience with many animated persons. These occasionally produced distracting noises, such as coughing or writing, and the fan in the projector could also be heard. The lecturer stood frontally (-15°) to the participant. A screen was placed next to him (25°), on which the slides of the lecture about a toolbox for the creation of acoustic scenes were shown. The speech of the lecturer was reproduced by two loudspeakers (51° and −38°). During the course of the lecture, a paper airplane flew over the heads of the audience and landed next to the lecturer. Target sound level L_eq_: 51.7 dBA, noise sound level L_eq_: 44.6 dBA.

The cafeteria VE was modelled after the cafeteria at the Carl von Ossietzky University Oldenburg Wechloy Campus. Here, the participant sat at a table with four animated people. Three of these people (-28°, 8°, 34°) talked to each other. In the background was cafeteria noise (diffuse recording of the Wechloy cafeteria, containing e.g. fragments of speech and plate clanging; Grimm and Hohmann, 2019), as well as music, laughter and conversations at the neighboring table. Target sound level L_eq_: 63.6 dBA, noise sound level L_eq_: 60.1 dBA.

The train station VE represented the Oldenburg central station. The participant stood on a platform and was asked to listen to the loudspeaker announcements, presented by several virtual loudspeakers, about trains arriving and departing. Meanwhile, trains were entering the station and animated persons were passing the participant with trolleys. On the neighboring platform, four people were talking to each other (98°). In addition, the beeping of a ticketing machine could be heard. Diffuse background noise (diffuse recording of Oldenburg central station) was present. Target sound level L_eq_: 68.3 dBA, noise sound level L_eq_: 67.8 dBA.

The street VE is another complex multi-talker situation. The noise here consisted of moving sound sources. The participant stood at a bus stop with four animated persons (-17°, 4°, 23°, 42°). Again, the task was to follow the conversation (hereafter: street_active_ VE). Meanwhile, vehicles drove past the participant on the right side. The moving sound sources consisted of trucks, cars, an ambulance with sounding siren, bicycles, talking passers-by and a singing mother with a baby carriage. In addition, children were playing in a schoolyard in the background and a train was passing by. As diffuse sound sources, birdsong and street noise were part of the scene (Grimm & Hohmann, 2019). Target sound level L_eq_: 63.2 dBA, noise sound level L_eq_: 63.4 dBA.

In addition to the street_active_ VE, a measurement with passive listening in a street VE (hereafter: street_passive_ VE) was carried out. Here, the participant stood at an intersection and had the task of waiting for a person to arrive with whom he had an appointment. The background noises were identical to those of the street_active_ VE. At the end of the scene, an animated person approached the participant.

## Appendix B

### Influence of Wearing Headphones and HARIR Channel Order Error

#### Method

An additional small experiment was conducted to test the influence of wearing headphones on self-motion, and the influence of the error in channel order for the preliminary HARIRs. For this experiment, 9 self-reported normal-hearing (NH) participants were recruited. Due to limitations imposed by the COVID-19 pandemic, participation in this experiment was limited to the authors and members of the research group “Auditory Signal Processing and Hearing Devices”. The task of the NH participants was to listen to the same VEs as the HI participants, once while the audio was played back over loudspeakers, and twice while the audio was played back over headphones (using preliminary and final HARIRs). For playback over loudspeakers, the aforementioned setup of 29 loudspeakers was used. For the NH participants, there was no individual adjustment of the noise level. The same movement measures as explained before were calculated on the head and gaze movement trajectories of the participants in this additional experiment, and a multiway ANOVA was done to compare the three listening conditions (headphones preliminary HARIRs, headphones final HARIRs, loudspeakers). As before, the false discovery rate was controlled with the Benjamini-Hochberg procedure (Benjamini & Hochberg, 1995).

### Results

The outcomes of the multiway ANOVA comparing the listening conditions are shown in Table B1. There was no significant effect of listening condition on any of the movement measures. This indicates that the effects of wearing headphones and channel order error are negligible for these measures.

**Table B1.** Main Effects of the Multiway ANOVA to Compare Listening Conditions: Headphones Final HARIRs (HP Final), Headphones Preliminary HARIRs (HP Prelim), Loudspeakers (LSPK).

Measure	95&per; Confidence Interval			F-value	p	p_adj_	Effect Size
	HP final	HP prelim	LSPK				
GazeStd	22.9–36.5	21.0–36.3	24.2–37.9	F(2,14) = 3.1	0.076	0.608	0.31
HeadStd	10.1–23.5	8.8–24.2	11.1–24.2	F(1.2,8.1) = 1.1	0.342	0.684	0.13
GazeSpeedMean	28.7–52.0	26.6–50.8	30.2–52.1	F(2,14) = 1.5	0.248	0.992	0.18
HeadSpeedMean	4.3–12.0	4.0–11.5	5.0–11.6	F(2,14) = 1.4	0.291	0.776	0.16
NGazeJumps	0.7–1.2	0.6–1.2	0.8–1.2	F(2,14) = 1.0	0.403	0.645	0.12
HeadGazeRatio	0.6–0.7	0.6–0.7	0.6–0.7	F(2,14) = 0.7	0.504	0.672	0.09
TargetGazeRMS	21.9–29.1	21.4–29.1	21.3–30.8	F(1.1,7.6) = 0.4	0.590	0.674	0.05
TargetHeadRMS	16.3–23.3	15.4–23.8	14.7–23.6	F(2,14) = 0.4	0.693	0.693	0.05

*Note.* The p_adj_-column lists p-values adjusted for multiple comparisons (Benjamini-Hochberg procedure). Significances are indicated at the *0.05 level **0.01 level ***0.001 level.
